# A systematic review and meta-analysis of the effects of resistance exercise on cognitive function in older adults

**DOI:** 10.3389/fpsyt.2025.1708244

**Published:** 2025-12-19

**Authors:** Jun Wu, Chuanfu Huang

**Affiliations:** 1 Department of Physical Education, Shanghai University of Engineering Science, Shanghai, China; 2 Department of Physical Education, Graduate School of Suwon University, Gyeonggi, Republic of Korea

**Keywords:** resistance training, cognitive function, older adults, meta-analysis, systematic review

## Abstract

**Objective:**

Cognitive decline has become a major concern with global population ageing, profoundly affecting quality of life and social participation in older adults. Resistance exercise has recently gained attention as a promising strategy to promote neuroplasticity and mitigate cognitive deterioration; however, evidence from randomized controlled trials (RCTs) remains inconsistent. This systematic review and meta-analysis aimed to evaluate the effects of resistance exercise on cognitive function in older adults and to examine whether improvements vary by age and whether exercise parameters—such as type, duration, session length, and weekly frequency—show dose–response relationships.

**Methods:**

PubMed, Web of Science, Cochrane Library, Embase, and Science Direct were systematically searched from database inception to September 2024 for RCTs investigating the effects of resistance training on cognitive function in older adults (≥60 years). Study quality was assessed using the Cochrane Risk of Bias 2 (ROB2) tool, and meta-analyses were conducted using RevMan 5.4 and Stata 17.

**Results:**

17 RCTs (n =739) met the inclusion criteria. Pooled analyses showed that resistance training significantly improved overall cognitive function (SMD = 0.40, P < 0.05), working memory (SMD = 0.44, P < 0.001), verbal learning and memory (MD = 3.01, P < 0.001), and spatial memory span (SMD = 0.63, P < 0.001), whereas effects on processing speed, executive function, and attention were not significant (P > 0.05). Heterogeneity and publication bias analyses indicated stable and unbiased results.

**Conclusion:**

Resistance exercise exerts selective benefits on cognitive domains in older adults, particularly enhancing overall cognition, working memory, verbal learning, and spatial memory. The magnitude of improvement appears to depend on age and exercise parameters, suggesting a potential dose–response relationship. These findings provide evidence-based guidance for resistance training into cognitive health promotion and rehabilitation programs for ageing populations.

**Systematic Review Registration:**

https://www.crd.york.ac.uk/prospero/, identifier CRD42023407397.

## 1 Introduction

Cognitive decline is a major public health concern accompanying global population ageing. As people age, older adults frequently experience impairments in memory, attention, information processing speed, and decision-making, which substantially increase the risk of developing cognitive disorders. According to the World Health Organization, approximately 46.8 million people worldwide were living with Alzheimer’s disease or dementia at the end of 2015, and this number is projected to rise to 74.7 million by 2030 and 131.5 million by 2050 (1). In China, an estimated 9.19 million individuals currently suffer from dementia, accounting for nearly 20% of the global total, with the number expected to exceed 30 million by 2050 (2). Therefore, identifying effective interventions to preserve and enhance cognitive function in older adults is critical for maintaining quality of life, delaying the onset of dementia, and alleviating societal and healthcare burdens.

Physical exercise has emerged as one of the most effective non-pharmacological strategies to delay age-related cognitive decline (3–6). Resistance exercise, also referred to as strength training, involves muscle contractions against external loads, such as body weight, dumbbells, barbells, elastic bands, or machines, to improve muscular strength and endurance. Recent studies have increasingly focused on the potential of resistance exercise to enhance cognitive function in older adults (7–9). For example, Best (10) reported that a one-year resistance training program led to improvements in working and spatial memory, accompanied by increases in brain volume. A previous review by Northey (3) conducted a systematic review indicating that resistance training positively affects overall cognition and may help prevent cognitive deterioration and brain atrophy. Liu (11)and Lindsay (12) demonstrated that resistance exercise not only enhances executive function in older adults but also significantly improves lower limb strength. Furthermore, Teresa (13) identified strength gains as a mediating factor in the positive effects of resistance exercise on cognitive function, suggesting improvements in overall cognition, attention, memory, and executive function. Cassilhas (14) observed superior performance on cognitive tests among older adults engaging in resistance exercise. Additionally, Nagamatsu (12) reported enhanced executive control, particularly in tasks requiring conflict resolution.

Although previous meta-analyses have consistently shown that physical exercise can significantly improve overall cognition, executive function, processing speed, and working memory in older adults (3, 15–17), important gaps remain. Specifically, the effects of resistance training on spatial memory span and verbal learning and memory—cognitive domains essential for independent living and social interaction—have received relatively little attention. In addition, prior reviews have not systematically examined whether training parameters, such as type, duration, session length, and weekly frequency, contribute to a dose–response relationship in cognitive outcomes. To address these gaps, the present study systematically synthesized randomized controlled trials that exclusively involved older adults without diagnosed mental disorders to examine the effects of resistance exercise on cognitive function. We incorporated additional outcomes, including spatial memory span and verbal learning, to provide comprehensive and quantitative evidence for clinical decision-making.

## 2 Subjects and methods

The study adhered to the PRISMA (Preferred Reporting Items for Systematic Reviews and Meta-Analyses) guidelines for systematic reviews and meta-analyses, as well as the requirements outlined in the Cochrane Handbook. It has been prospectively registered in the International Prospective Register of Systematic Reviews (PROSPERO) under registration number No. CRD42023407397.

### 2.1 Literature search strategy

A computerized search was conducted in the PubMed, Web of Science, Science Direct, and Embase databases to identify randomized controlled trials (RCTs) evaluating the effects of resistance training on cognitive function in older adults. The search spanned from the inception of each database to September 2024 (**Supplementary Material 1**). To ensure comprehensive coverage, additional relevant studies were identified through manual searches of related journals, reference lists of included studies, and existing systematic reviews. The search strategy combined MeSH terms and free-text keywords; the English search terms used were::resistance exercise, strength training, weight exercise, cognitive function, cognitive performance, executive function, inhibition, task switching, working memory, oldest, older adults, elderly, geriatric, aging, older people.

Following the search, two researchers independently screened the literature in a double-blind process according to the inclusion and exclusion criteria. First, references were imported into EndNote X20 software for deduplication. Titles and abstracts were reviewed for preliminary screening, and full texts of potentially eligible studies were retrieved and thoroughly assessed. After screening, the two researchers compared the selected studies, and any disagreements were resolved through discussion with a third researcher to reach a consensus on inclusion.

### 2.2 Inclusion and exclusion criteria

Inclusion Criteria: (1) Participants aged 60 years or older; (2) intervention method focused on resistance training; (3) primary or secondary outcome measures included cognitive function; and (4) study design classified as experimental research.

Exclusion Criteria: (1) Participants diagnosed with mild cognitive impairment, dementia, neurological disorders, or mental health disorders; (2) interventions incorporating non-exercise confounding factors, such as cognitive training, vitamin supplements, or medication; (3) studies for which data were unavailable despite contacting authors; and (4) qualitative studies, case studies, reviews, non-interventional studies, and conference abstracts.

### 2.3 Data extraction and coding

Two researchers independently extracted and coded relevant information from included studies. Extraction and coding details included: (1) basic study information: author, country, and publication year; (2) participant information: age, gender, education level, and sample size; (3) exercise variables: categorized according to prior research findings; and (4) outcome measures: cognitive function in older adults, extracted as mean and standard deviation values.

### 2.4 Quality assessment of included studies

The risk of bias in included studies was assessed according to the Cochrane Handbook for Systematic Reviews of Interventions, version 6.3 (2022). Evaluation items included: (1) bias arising from the randomization process; (2) bias due to deviations from intended interventions; (3) bias from missing outcome data; (4) bias in the measurement of outcomes; and (5) bias from selective reporting of results. Two researchers independently rated each item’s signaling questions as "Y" (Yes), "PY" (Probably Yes), "N" (No), "PN" (Probably No), or "NI" (Not Reported). The quality assessment categorized studies as high risk, low risk, or some concerns of bias. The Revised Cochrane risk-of-bias tool for randomized trials (ROB2) was used to summarize risk across subdomains and provide an overall risk of bias rating for each included study (18).

The GRADEpro system (19) was also used to assess the quality of evidence for each outcome measure, categorizing evidence quality into four levels: high, moderate, low, and very low. Quality assessments were independently conducted by two researchers, with any discrepancies resolved through discussion with a third researcher until consensus was reached.

### 2.5 Statistical analysis

Meta-analyses were conducted using RevMan 5.4.1 to pool effect sizes, assess heterogeneity, and perform subgroup analyses. Sensitivity analyses and publication bias were evaluated using Stata 17.0. All outcome data were continuous variables. When different studies assessed the same cognitive domain using identical measurement methods and units, the mean difference (MD) was used; when measurement methods or units differed, the standardized mean difference (SMD) was calculated. If studies did not report standard deviations (SDs), these were derived from standard errors (SEs), confidence intervals (CIs), or t-values. For outcomes where higher values indicated worse cognitive performance (e.g., reaction time, error counts), means were multiplied by −1 to ensure consistency in the direction of effect.

For studies reporting multiple outcomes within the same cognitive domain or at different time points, a single primary outcome was selected for the main analysis to avoid double-counting. When multiple time points were available, the endpoint measure was prioritized. Sensitivity analyses including all available outcomes were conducted to assess the robustness of the findings. Effect sizes were reported with point estimates and 95% CIs. Heterogeneity was assessed using the I² statistic and the P-value; I² > 50% or P < 0.05 indicated substantial heterogeneity, in which case a random-effects model was applied, otherwise a fixed-effects model was used. Statistical significance between intervention and control groups was considered at P < 0.05.

## 3 Results

### 3.1 Study inclusion

**Figure 1:**
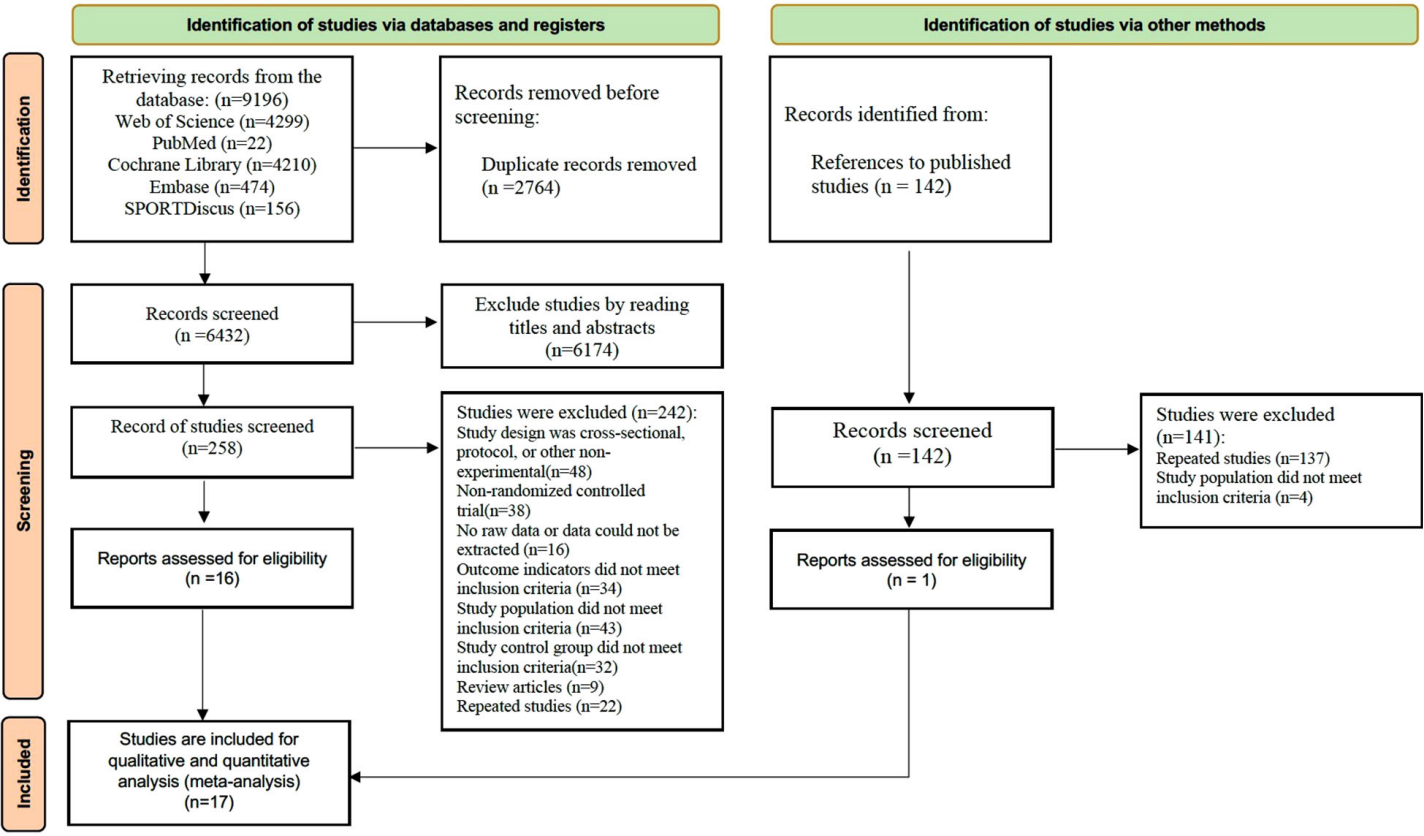
PRISMA 2020 flow diagram illustrating the study selection process.

A total of 9,196 records were initially identified from five electronic databases (**Figure 1**): Web of Science (n = 4,299), PubMed (n = 22), Cochrane Library (n = 4,210), Embase (n = 474), and SPORTDiscus (n = 156). After importing all records into EndNote X20 and removing duplicates (n = 2,764), 6,432 unique records remained for title and abstract screening. Following this preliminary screening, 6,174 records were excluded for irrelevance to the study topic, leaving 258 articles for full-text assessment. Of these, 242 were further excluded for the following reasons: cross-sectional or non-RCT design (n = 18), non-randomized controlled trial (n = 38), unavailable data (n = 16), outcomes not meeting the inclusion criteria (n = 34), study population not meeting the inclusion criteria (n = 43), control group not meeting the inclusion criteria (n = 32), review articles (n = 9), and duplicate publications (n = 22).In addition, 142 records were identified through other sources, including reference lists of relevant studies and previously published reviews. After screening, 141 records were excluded (duplicates, n = 137; study population not meeting inclusion criteria, n = 4), leaving one additional article for full-text evaluation. Ultimately, 17 randomized controlled trials (RCTs) were included in the qualitative and quantitative synthesis (meta-analysis).

A total of 17 randomized controlled trials (RCTs) were included in this study, published between 1998 and 2024, with a combined sample size of 739 participants (360 in the experimental group and 379 in the control group). Participants were primarily recruited from community settings, with an average age ranging from 62.4 to 87.6 years. All included studies focused solely on resistance training, employing exercise modalities such as variable resistance training (using resistance bands) and conventional resistance training (**Table 1**). The duration of the exercise interventions ranged from 6 to 24 weeks, with frequency varying from 1 to 3 times per week, most commonly at 3 times per week. The duration of each intervention session was between 30 and 60 minutes, with a notable concentration around 60 minutes. The control groups maintained their usual daily activities or received no intervention. Outcome measures primarily included overall cognitive function, information processing speed, working memory, executive function, verbal learning and memory, visual learning and memory, attention, and spatial memory span (**Table 2**).

**Table 1:** Basic information of studies included in the meta-analysis.

Study	Country	Sample size(E/C)	Gender(M/F)	Age (E/C)	Exercise type	Intervention dose				Control group	Outcome measures
						Time (min)	Frequency (times per week)	Cycle (week)	Intensity		
André (20)(2016)	Brazil	8/29	0/37	66.8/66.8	Resistance Exercise	60	3	12	60%-70%1RM	Daily activities	MoCA
Paulo (21)(2020)	Brazil	24/25	64%/60%	66/66	Resistance Exercise	50-60	3	12	/	Daily activities	MoCA, ST, DSF, DSB, VFT
Miguel (22)(2022)a	Spain	20/19	0/39	87.6/81.3	Elastic Band	50	3	12	Moderate	Stretching training	MMSE, TMT, Fototest
Miguel (22)(2022)b	Spain	29/19	0/39	81.4/81.3	Elastic Band	50	3	12	Moderate	Stretching training	MMSE, TMT, Fototest
Humberto (23)(2021)	Chile	14/15	/	69.89	Resistance Exercise	60	3	12	50%-60%1RM	Daily activities	MMSE
Petrosya (24)(2013)	USA	12/12	24/0	66.7/67.4	Resistance Exercise	50-60	3	12	50%-60%1RM	Daily activities	IFR, DFR, VLMT
Pasquauna (25)(1998)	Switzerland	23/23	28/18	65-69	Resistance Exercise	/	1	8	/	Daily activities	IFR, DFR, WAIS
Enzo (26)(2015)	Italy	20/20	17/23	65.8/66.4	Resistance Exercise	30	3	12	60%-85%1RM	Daily activities	AM, RPM, SCWI, TMT, DCT
Maren (27)(2014)	USA	13/12	/	70.6/70.6	Resistance Exercise	/	2	6	OMNI:5-6	Daily activities	3D-MOT
Carla (28)(2017)	South Africa	22/19	15/26	62.4/62.5	Resistance Exercise	40	3	16	50%-100%10RM	Daily activities	ST
Hélio (29)(2020)a	Brazil	10/14	0/24	67/66.7	Resistance Exercise	60	2	24	RPE:3–4 points	Daily activities	MMSE, SMT
Hélio (29)(2020)b	Brazil	12/14	0/26	66.7/66.7	Elastic Band	60	2	24	RPE:3–4 points	Daily activities	MMSE, SMT
Ricardo (14)(2007)a	Brazil	19/23	42/0	69/67	Resistance Exercise	60	3	24	50%1RM	Daily activities	WAIS, WMS-R, TP-S, ROCF
Ricardo (14)(2007)b	Brazil	20/23	42/0	68.4/67	Resistance Exercise	60	3	24	80%1RM	Daily activities	WAIS, WMS-R, TP-S, ROCF
Juliana (30)(2015)	Brazil	23/23	14/32	82.8/82.6	Resistance Exercise	60	3	16	BORG:14–17 points	Daily activities	MoCA
Chen (31)(2023)	USA	14/14	6/22	76.8/77.6	Resistance Exercise	50	2	12	BORG :13–16 points	Daily activities	MoCA
Natan (32)(2023)	Brazil	19/18	20/17	68/73.4	Resistance Exercise	/	2	12	70%1RM	Daily activities	Trail Making test, Digit Span Test
Corbin (33)(2024)	UK	18/18	26/0	67/63	Resistance Exercise	/	2	12	80%1RM	Daily activities	Reaction time, Multitasking test, Domain-specific z-scores
Junga (34)(2024)	Korea	13/14	10/17	70/71.5	Resistance Exercise	30	3	8	/	Daily activities	Reaction time, Multitasking test, Domain-specific z-scores
Wouter (35)(2024)	Netherlands	27/25	28/24	69/70.7	Resistance Exercise	/	2	12	/	Daily activities	MoCA, ANAM test, Go/No-go test

E, Experimental group; C, Control group; M, Male; F, Famle; RM, Repetitions Maximum; MoCA, Montreal Cognitive Assessment; MMSE, Mini-Mental State Examination; ST, Stroop Test Incongruent/Stroop Interference; DSF, Digit Span Forward; DSB, Digit Span Backward; VFT, Verbal Fluency Test; TMT, Trail Making Test; Fototest, Photo Test; IFR, Immediate Free Recall; DFR, Delayed Free Recall; VLMT, Verbal Learning and Memory Test; WAIS, Wechsler Adult Intelligence Scale; DST, Digit-Symbol Test; AM, Attentive Matrices Test; RPM, Raven’s Progressive Matrices; SCWI, Stroop Color and Word Test; DCT, Drawing Copy Test; 3D-MOT, 3D Multiple Object-Tracking; SMT, Short-Term Memory Test; WMS-R, Wechsler Memory Scale-Revised; TP-S, Toulouse-Pieron-S Attention Test; ROCF, Rey-Osterrieth Complex Figure Test. /: Not specified.

**Table 2:** Overview of cognitive functions and associated tasks.

Cognitive function	Cognitive task
Overall Cognitive Function	MoCA, MMSE, RPM, Fototest
Information Processing Speed	DST, TMT, ST
Working Memory	SMT, DFR, IFR, DST
Executive Function	ST,
Verbal Learning and Memory	VLMT
Visual Learning and Memory	DCT, 3D-MOT, ROCF
Attention	AM, TP-S
Spatial Memory Span	ROCF

### 3.2 Risk of bias assessment

**Figure 2:**
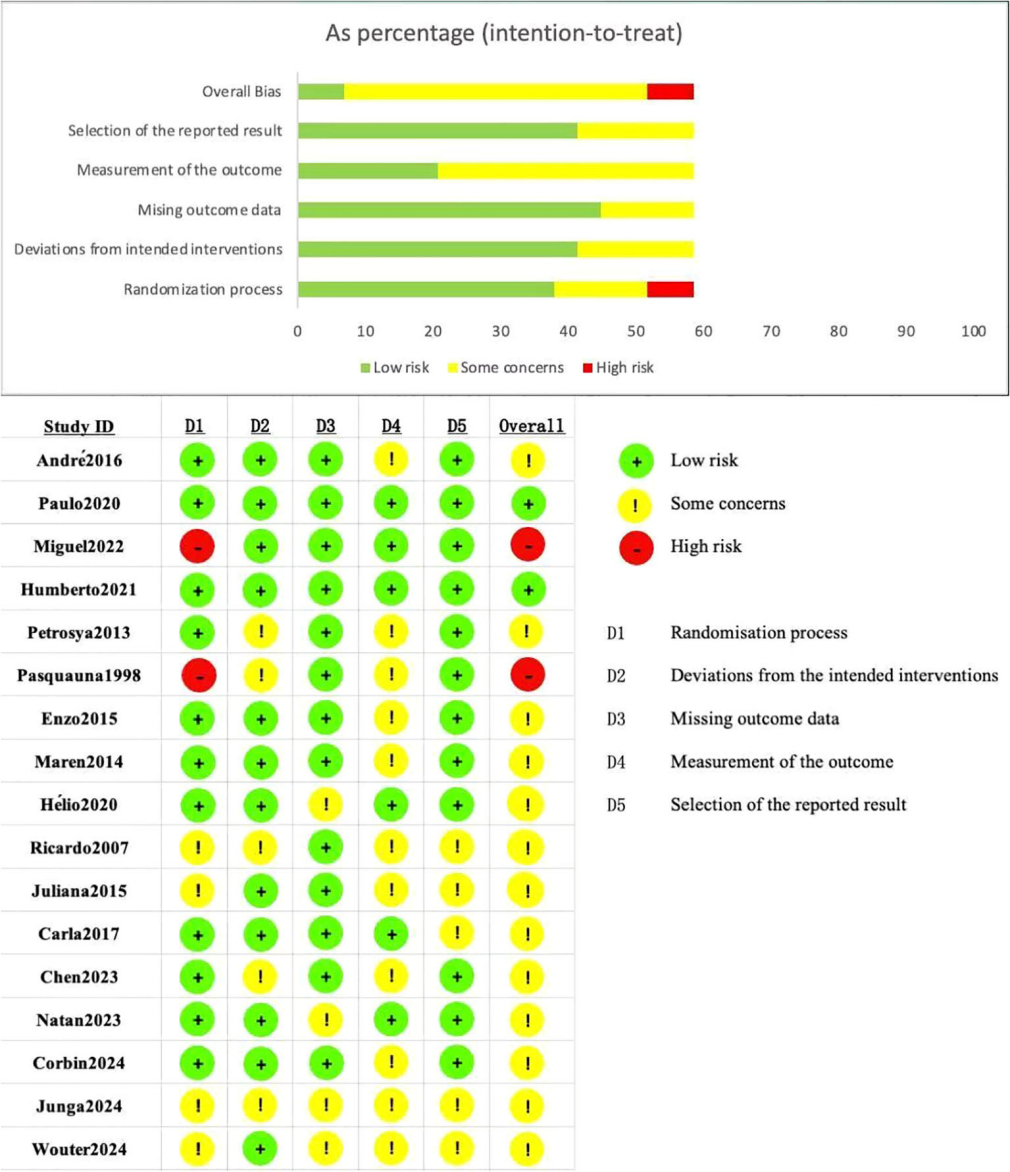
Risk of bias assessment for included studies. Each study shown in the Risk of Bias Assessment for Included Studies is identified by the first author’s name and publication year, and all corresponding references are listed in the main reference section.

This study included 17 randomized controlled trials (RCTs) for meta-analysis. The risk of bias was summarized for each subdimension using the Cochrane Risk of Bias tool for randomized trials (ROB2) (**Figure 2**). Among these studies, 11 provided detailed descriptions of the random sequence generation methods, indicating a low risk of bias. Twelve studies specifically reported the anticipated interventions, also reflecting a low risk of bias. Thirteen studies showed no evidence of bias due to missing outcome data, while 6 studies demonstrated no bias in outcome measurement. Regarding selective reporting bias, 12 studies were classified as having a low risk of bias.

### 3.3 Meta-analysis results

#### 3.3.1 Effect of resistance training on information processing speed in older adults

**Figure 3:**
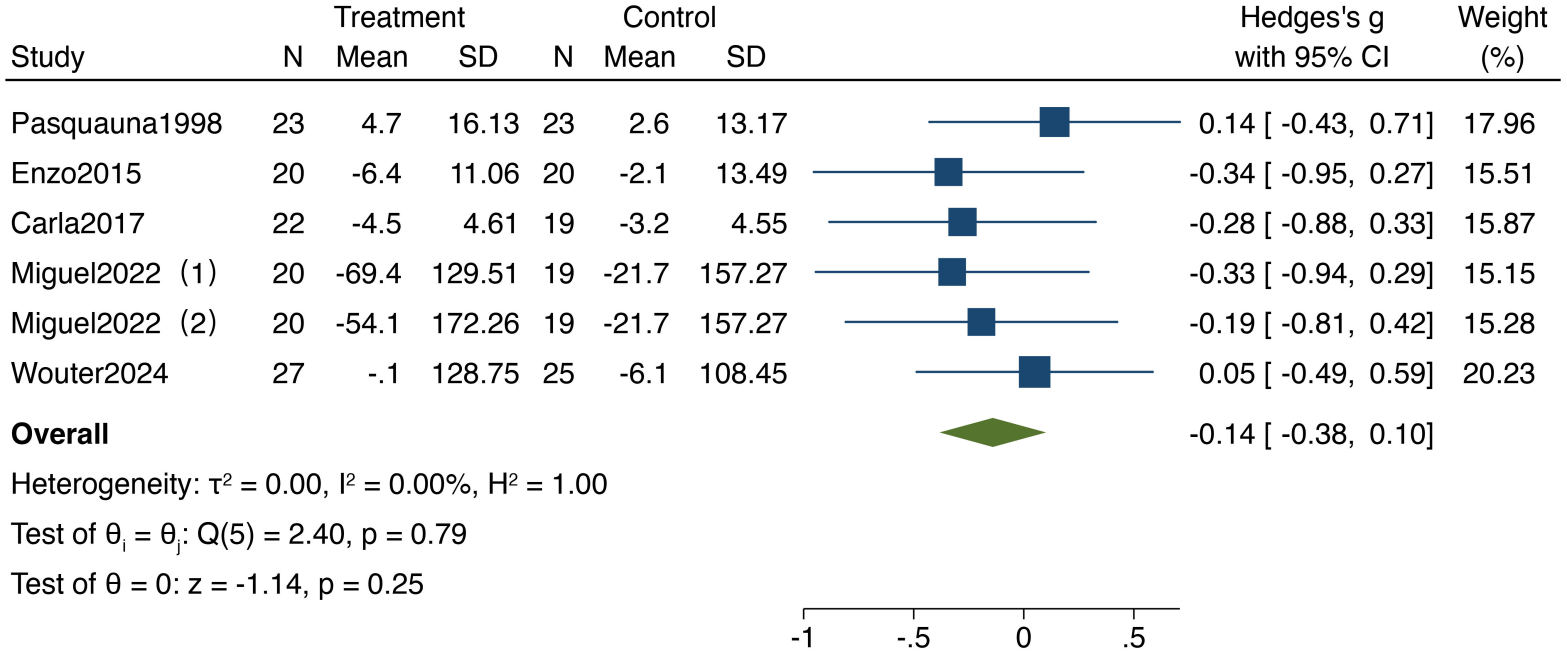
Forest plot of meta-analysis for information processing speed. Each study shown in the forest plot is identified by the first author’s name and publication year, and all corresponding references are listed in the main reference section.

As illustrated in **Figure 3**, five studies included in the analysis reported on the impact of resistance training on information processing speed in older adults, encompassing a total of 257 participants (132 in the experimental group and 125 in the control group).The analysis was characterized by low statistical heterogeneity (I² = 0%,95%CI: 0%,61%,P = 0.79) though the imprecision of this estimate is reflected in the wide confidence interval. The results demonstrated a standardized mean difference (SMD) of -0.14 (95% CI: -0.38,0.10,P = 0.25), indicating no significant improvement in information processing speed following resistance training, with no notable differences compared to the control group.

#### 3.3.2 Effect of resistance training on working memory in older adults

**Figure 4:**
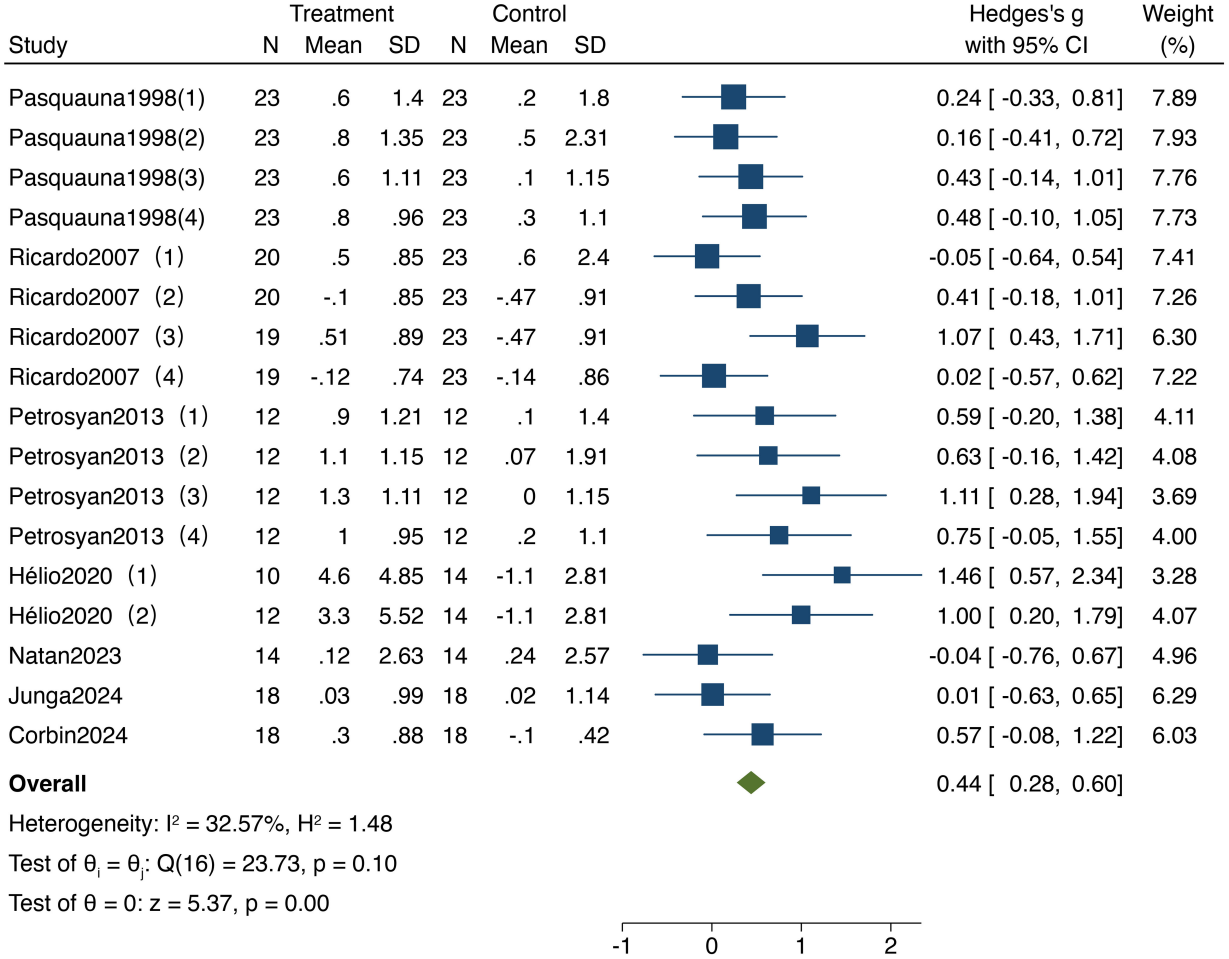
Forest plot of meta-analysis for working memory. Each study shown in the forest plot is identified by the first author’s name and publication year, and all corresponding references are listed in the main reference section.

As shown in **Figure 4**, 7 studies included in the analysis reported on the impact of resistance training on working memory in older adults, encompassing a total of 600 participants (290 in the experimental group and 310 in the control group). The heterogeneity test results (I² = 32.57%,95%CI: 0%,61.4%,P = 0.10) indicated no significant heterogeneity; therefore, a fixed-effect model was employed for analysis. The results revealed a standardized mean difference (SMD) of 0.44 (95% CI: 0.28, 0.60, P < 0.001), indicating a significant improvement in working memory following resistance training, with notable differences compared to the control group.

#### 3.3.3 Effect of resistance training on executive function in older adults

**Figure 5:**
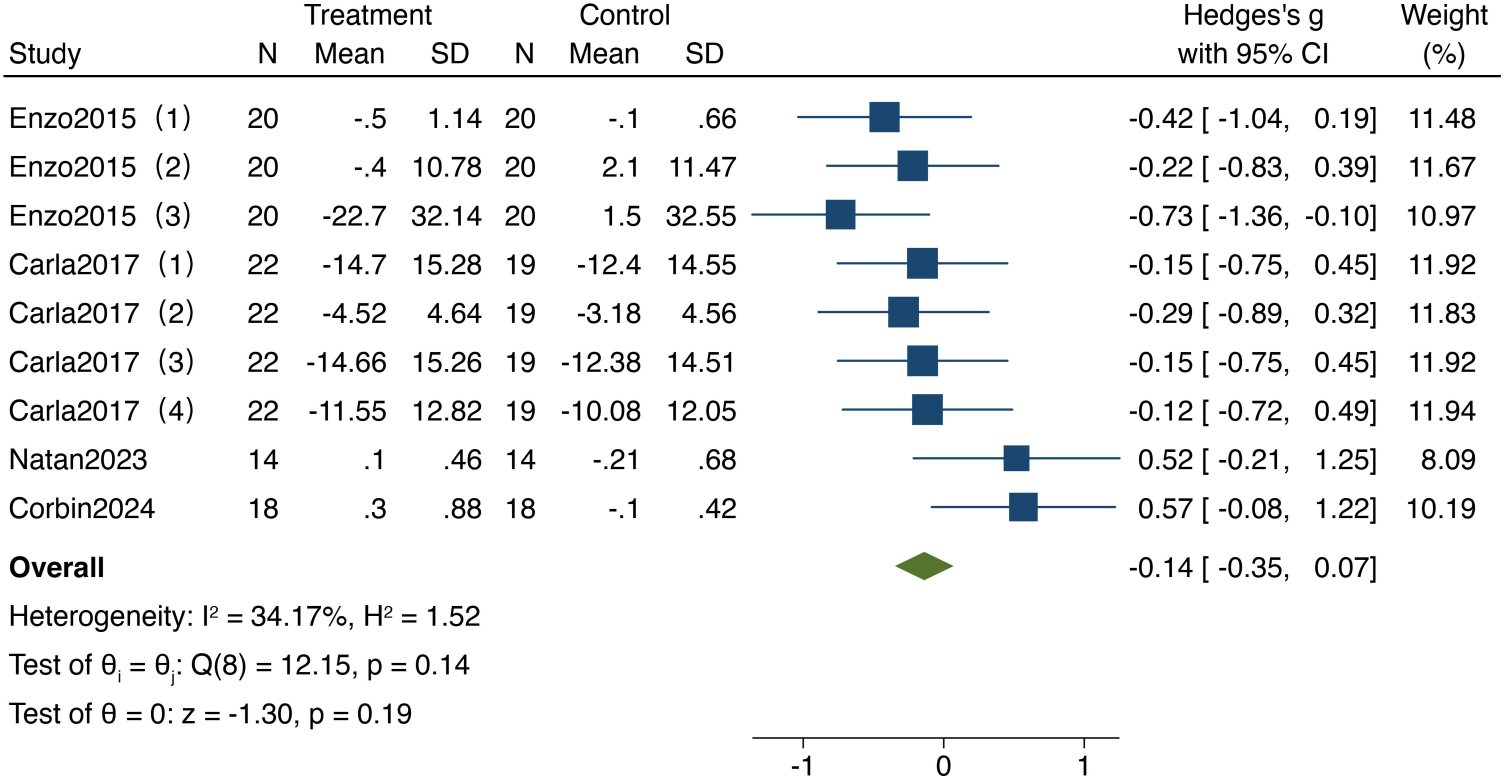
Forest plot of meta-analysis for executive function. Each study shown in the forest plot is identified by the first author’s name and publication year, and all corresponding references are listed in the main reference section.

As illustrated in **Figure 5**, 4 studies included in the analysis reported on the impact of resistance training on executive function in older adults, involving a total of 366 participants (180 in the experimental group and 168 in the control group). The heterogeneity test results (I² = 34.17%,95%CI: 0%,68.5%,P = 0.14) indicated no significant heterogeneity; therefore, a fixed-effect model was utilized for the analysis, while also exploring potential sources of heterogeneity. The results demonstrated a standardized mean difference (SMD) of -0.14 (95% CI: -0.35, 0.07, P = 0.19), indicating no significant differences in executive function following resistance training compared to the control group.

#### 3.3.4 Effect of resistance training on attention in older adults

**Figure 6:**
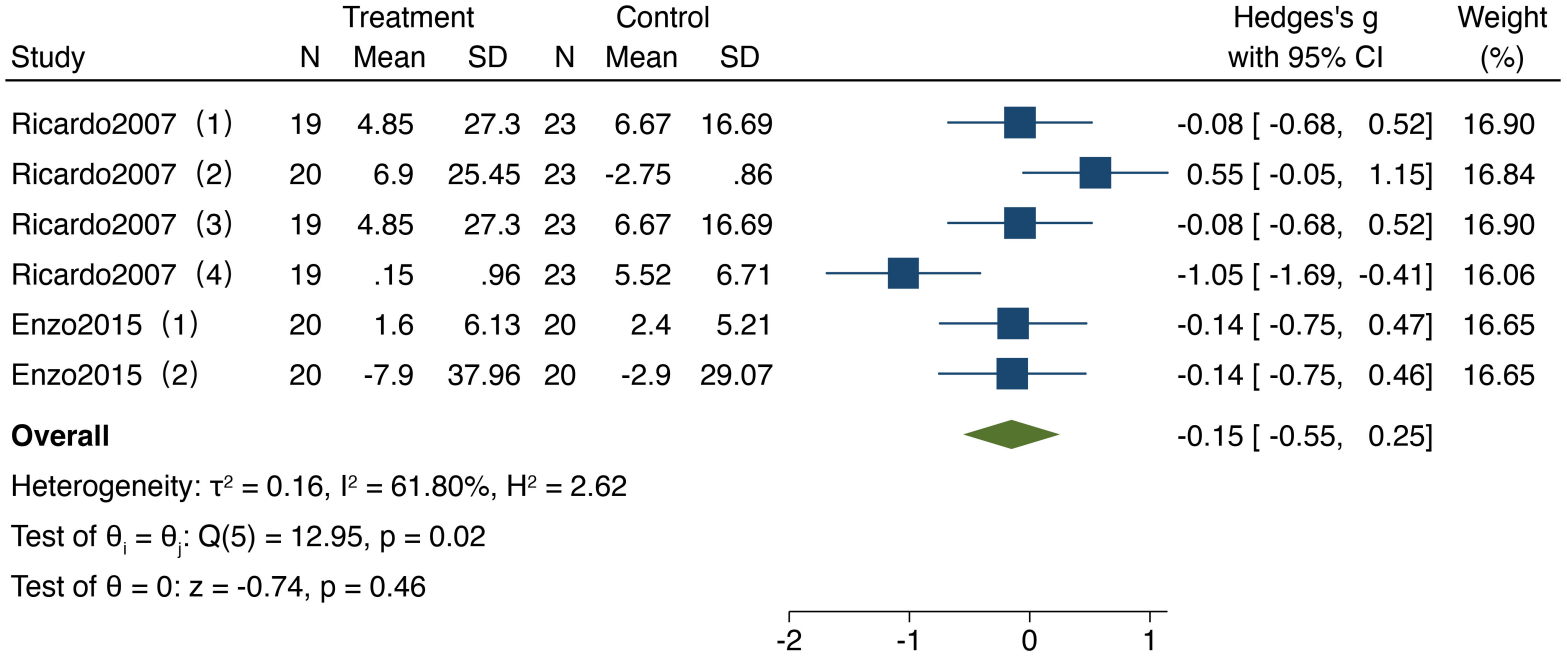
Forest plot of meta-analysis for attention. Each study shown in the forest plot is identified by the first author’s name and publication year, and all corresponding references are listed in the main reference section.

As depicted in **Figure 6**, 2 studies included in the analysis examined the impact of resistance training on attention in older adults, involving a total of 249 participants (117 in the experimental group and 132 in the control group). The heterogeneity test results (I² = 61.8%,95%CI:0%,82.1%, P = 0.02) indicated the presence of heterogeneity; thus, a fixed-effect model was applied for the analysis. The results revealed a standardized mean difference (SMD) of -0.15 (95% CI: -0.55, 0.25, P = 0.46), indicating no significant differences in attention following resistance training compared to the control group.

#### 3.3.5 Effect of resistance training on spatial memory span and verbal learning and memory in older adults

Due to the limited literature available—only one study each addressing the effects of resistance training on spatial memory span and verbal learning and memory—forest plot analyses were not performed for these outcomes. One study reported the effect of resistance training on spatial memory span in older adults, involving a total of 252 participants (114 in the experimental group and 138 in the control group). The effect size for this study was a standardized mean difference (SMD) of 0.63 (95% CI: 0.29, 0.96, P = 0.0009), indicating a significant improvement in spatial memory span following resistance training compared to the control group. In another study, the impact of resistance training on verbal learning and memory was assessed, including 48 older adults (24 in the experimental group and 24 in the control group). The effect size for this outcome was a mean difference (MD) of 3.01 (95% CI: 2.38, 3.64, P < 0.001), demonstrating a significant improvement in verbal learning and memory post-intervention compared to the control group.

#### 3.3.6 Effect of resistance training on overall cognitive function in older adults

**Figure 7:**
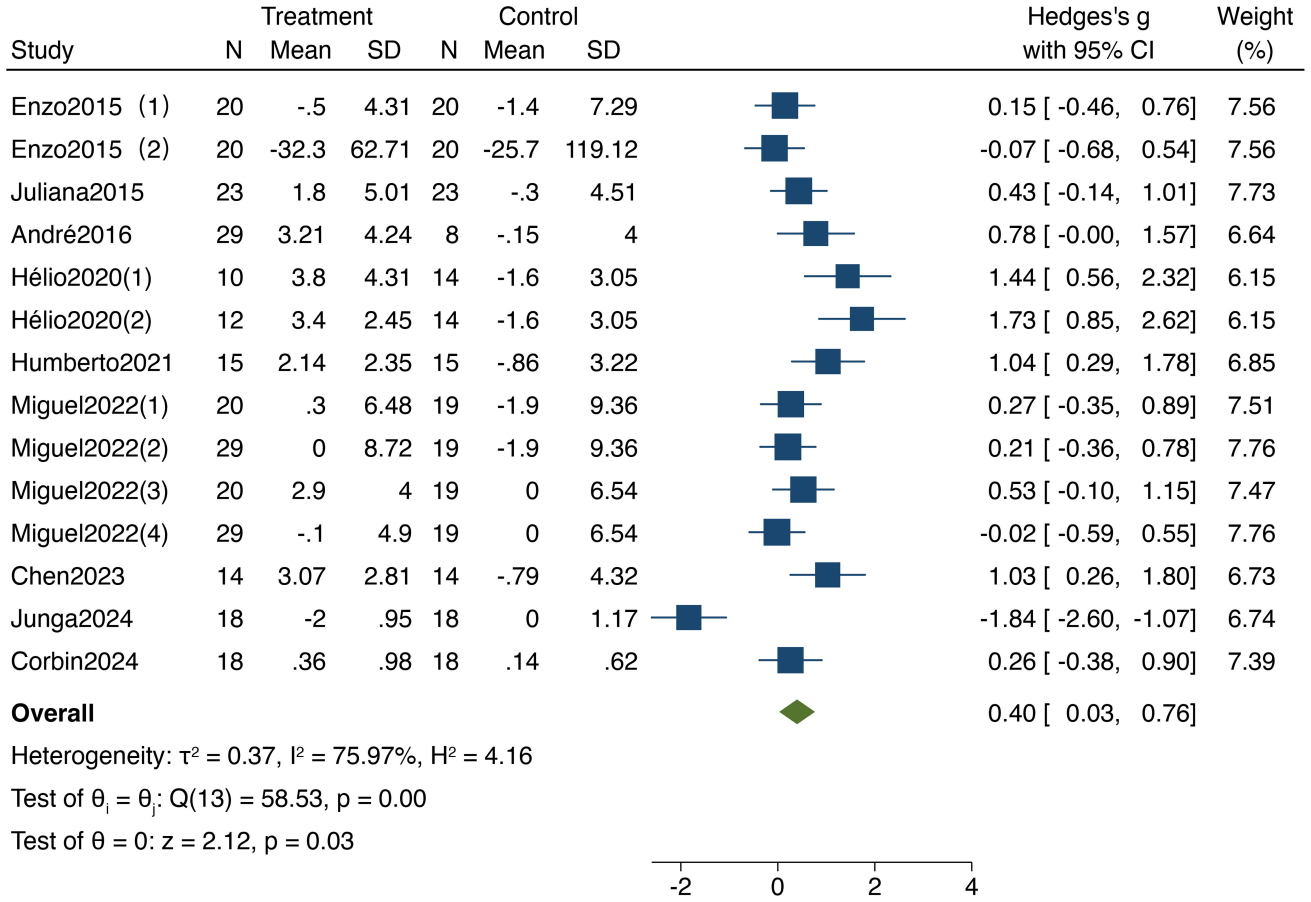
Forest plot of meta-analysis for overall cognitive function. Each study shown in the forest plot is identified by the first author’s name and publication year, and all corresponding references are listed in the main reference section.

As shown in **Figure 7**, 9 studies included in the analysis examined the impact of resistance training on overall cognitive function in older adults, encompassing a total of 517 participants (277 in the experimental group and 240 in the control group). The heterogeneity test results (I²=75.97%,95%CI:60%,85.3%,P < 0.05) indicated significant heterogeneity, leading to the use of a random-effects model for the analysis. The results demonstrated a standardized mean difference (SMD) of 0.40 (95% CI: 0.03, 0.76, P < 0.05), indicating a significant enhancement in overall cognitive function following resistance training compared to the control group.

#### 3.3.7 Subgroup analysis of the effects of the five components of exercise on overall cognitive function in older adults

This study conducted subgroup analyses of cognitive function research characterized by high heterogeneity, examining factors such as age group, exercise duration, exercise type, exercise frequency, and exercise duration. As shown in **Table 3**, the included studies were categorized into two age subgroups: 65–74 years and those over 74 years. For exercise duration, the studies were divided into subgroups of 12 weeks and more than 12 weeks. Regarding exercise type, the studies were classified into variable resistance exercise and traditional resistance exercise subgroups. For exercise frequency, the groups included those engaging in exercise three times per week and two times per week. Finally, for exercise duration, the studies were divided into two subgroups: 30–50 minutes and 60 minutes.

**Table 3:** Subgroup analysis of the effects of resistance exercise on overall cognitive function in older adults.

Characteristics	Group	Number of included studies (sample size)	MD	95%CI	*P*	*I^2^/%*	*P_Heterogeneity_*
Age	65-74	14(576)	0.27	-0.06, 0.61	<0.00001	73	0.11
	>75	3(248)	0.35	0.10, 0.61	0.41	0	0.007
	Total	17(824)	0.30	0.06, 0.55	<0.00001	54	0.02
Cycle	12weeks	8(465)	0.35	0.15, 0.56	0.29	16	0.0008
	>12weeks	3(132)	0.43	-1.07, 1.93	<0.00001	93	0.57
	Total	11(597)	0.39	0.06, 0.73	<0.00001	54	0.02
Type	Variable Resistance	5(200)	0.46	-0.02, 0.94	0.06	63	0.03
	Resistance Exercise	10(397)	0.33	-0.12, 0.77	<0.00001	78	0.15
	Total	15(597)	0.50	0.20, 0.81	<0.00001	54	0.02
Frequency	3 times/week	6(403)	0.16	-0.25, 0.56	<0.00001	64	0.45
	Twice/week	5(194)	0.77	0.24, 1.29	0.01	67	0.005
	Total	11(597)	0.37	0.05, 0.70	<0.00001	54	0.02
Time	30min-50min	4(318)	0.04	-0.44, 0.52	<0.00001	57	0.85
	60min	4(89)	1.01	0.54, 1.47	<0.0001	43	0.13
	Total	8(481)	0.41	-0.00, 0.82	<0.00001	54	0.02

The results of the subgroup analyses indicated that the heterogeneity of the combined effect sizes for age, exercise duration, exercise type, exercise frequency, and exercise duration remained consistent at 54%, showing no change compared to the overall combined effect size, suggesting that these factors were not sources of heterogeneity. Specifically, a significant improvement effect was observed in the 65–74 year age group (P < 0.001), and a certain degree of dose-response effect was noted in relation to the intervention type, duration, frequency, and duration (P < 0.001).

Among the included studies, five reported objective exercise intensities (60%-70% 1RM, 60%-85% 1RM, 50%-60% 1RM), two reported subjective ratings of perceived exertion (RPE 3-4, BORG 14-17), and one study did not report the exercise intensity. Due to the inconsistency in the evidence regarding exercise intensity, subgroup analysis based on this factor was not performed.

### 3.4 Sensitivity analysis

To investigate whether the observed heterogeneity among studies was due to a single study, we conducted a sensitivity analysis on the effect of resistance training on overall cognitive function in older adults (**Table 4**). By sequentially excluding each individual study, we reassessed the combined effect size, as shown in **Table 4**. The range of the combined effect size (SMD) was from -1.84 to 4.47, with I² values ranging from 87% to 91%, and all P values were less than 0.001.

**Table 4:** Sensitivity analysis of the effect of resistance exercise on overall cognitive function in older adults.

Study	SMD	95%CI	P	I^2^(%)
André2016	0.78	-0.02,1.59	<0.001	91
Miguel2022	4.47	3.25,5.69	<0.001	88
Miguel2022	3.79	2.81,4.78	<0.001	87
Miguel2022	0.21	-0.37,0.79	<0.001	91
Miguel2022	0.27	-0.36,0.9	<0.001	91
Humberto2021	1.04	0.27,1.8	<0.001	91
Enzo2015	-0.07	-0.69,0.55	<0.001	91
Enzo2015	0.15	-0.47,0.77	<0.001	91
Hélio2020	1.44	0.51,2.37	<0.001	91
Hélio2020	1.73	0.81,2.66	<0.001	91
Juliana2015	0.43	-0.15,1.02	<0.001	91
Chen2023	1.03	0.23,1.82	<0.001	91
Griffen2024	0.26	-0.39,0.92	<0.001	91
Lee2024	-1.84	-2.63,-1.04	<0.001	88

### 3.5 Publication bias

**Figure 8:**
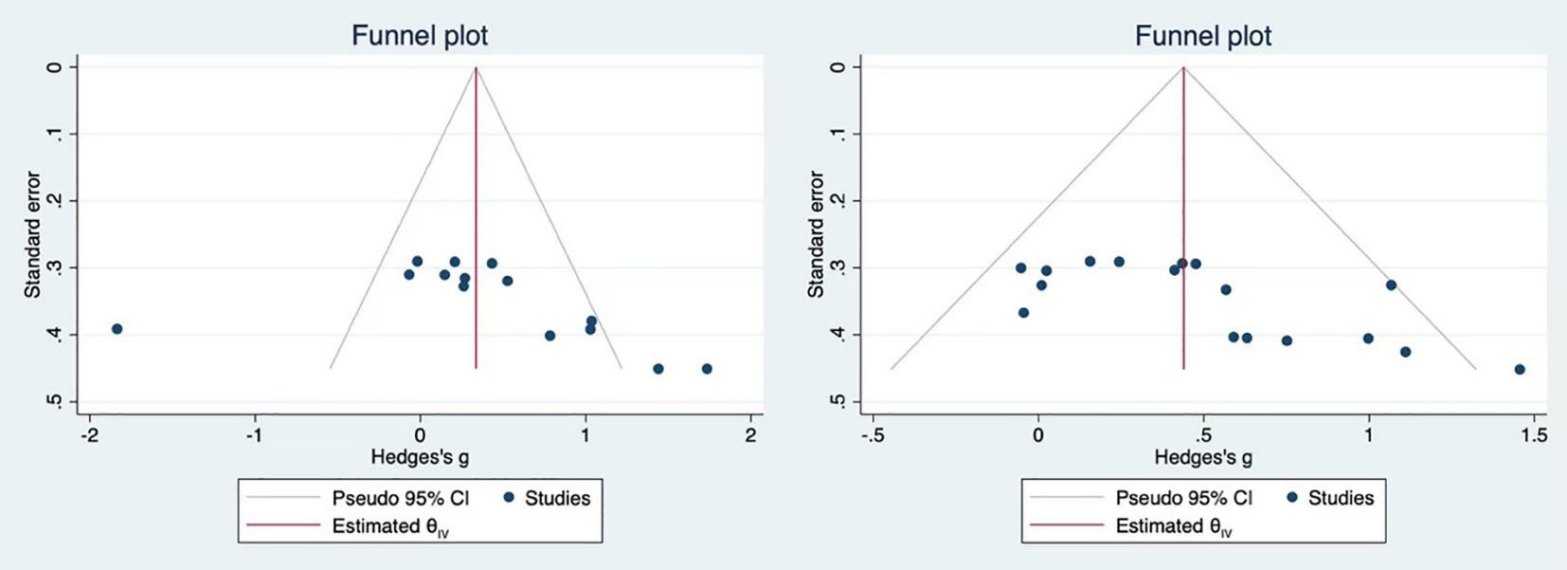
Funnel plot for overall cognitive function and working memory.

As shown in **Figure 8**, publication bias was assessed for the effects of resistance training on overall cognitive function and working memory in older adults. The Egger’s test results were as follows: Overall cognitive function (t = 1.90, P = 0.0576 > 0.05), These results indicate that no publication bias was present; Working memory, (t = 3.12, P = 0.0018 < 0.05), suggesting a presence of publication bias in the studies. Trim-and-fill analysis was performed on the left side of the funnel plot, yielding an adjusted effect size of 0.439 with a 95% CI [0.279 to 0.599]. Therefore, it was advised to interpret the results of the working memory cautiously due to the identified publication bias.

### 3.6 Quality of evidence evaluation

As shown in **Table 5**, the 17 studies included in this review were of relatively moderate quality. Subgroup and sensitivity analyses were conducted on the effects of resistance training on overall cognitive function in older adults, but no sources of heterogeneity were identified, indicating substantial limitations. The evidence quality evaluation results revealed that the effects of resistance training on overall cognitive function and working memory in older adults were of moderate quality. The remaining outcome indicators were of low quality.

**Table 5:** Evidence quality assessment.

Outcomes	RCTs	Participants	Effect size	95%CI	Quality of evidence
Information Processing Speed	5	257	-0.14	-0.38,0.10	low
Working Memory	7	600	0.44	0.28,0.60	moderate
Executive Function	4	366	-0.14	-0.35,0.07	low
Attention	2	249	-0.15	-0.55,0.25	low
Overall Cognitive Function	9	517	0.4	0.03,0.76	moderate

GRADE Working Group grades of evidence High quality: Further research is very unlikely to change our confidence in the estimate of effect. Moderate quality: further research is likely to have an important impact on our confidence in the estimate of effect and may change the estimate. Low quality: Further research is very likely to have an important impact on our confidence in the estimate of effect and is likely to change the estimate. Very low quality: We are very uncertain about the estimate.

### 3.7 Adverse events

None of the 17 studies included in this review reported adverse events resulting from exercise interventions.

## 4 Discussion

In this systematic review and meta-analysis, 9,196 records were identified from five major electronic databases, with an additional 142 records from other sources. After removing duplicates and screening titles, abstracts, and full texts, 17 randomized controlled trials (RCTs) were included in the qualitative and quantitative synthesis. A total of 17 studies were included in this study to systematically evaluate and analyze the effects of resistance training on cognitive function in older adults. Studies assessed multiple cognitive domains, and continuous outcomes were analyzed using mean differences (MDs) or standardized mean differences (SMDs), according to measurement consistency. Any discrepancies in study selection were resolved through consultation with a third-party expert to ensure reliability. This review's strengths include a comprehensive search strategy, pre-specified inclusion and exclusion criteria, independent dual screening, and standardized data extraction and effect size calculation. Sensitivity analyses and publication bias assessments further reinforced the robustness of the findings. Harmonization of effect directions across studies minimized potential biases from outcome reporting heterogeneity. Study quality and risk of bias were evaluated using GRADEPro and ROB2, showing generally high methodological quality. The overall quality of the included literature was relatively good. Some heterogeneity was observed, likely due to selective reporting of blinding procedures, unclear outcome measurement methods, or variability in outcome measurement. However, subgroup and sensitivity analyses conducted on the effects of resistance training on overall cognitive function in older adults did not identify the sources of heterogeneity. Publication bias was assessed for both overall cognitive function and working memory. For overall cognitive function, Egger's test indicated no significant publication bias. In contrast, for working memory, Egger's test suggested the possible presence of publication bias. Therefore, a trim-and-fill analysis was performed, which showed that the adjusted effect size remained statistically significant, although the point estimate was somewhat reduced. This finding indicates that the initial analysis might have overestimated the true effect of resistance training on working memory, and the original results should be interpreted with caution. The evidence quality assessment rated the evidence for the effects of resistance training on both overall cognitive function and working memory in older adults as moderate. For the remaining outcome indicators, the evidence quality was low, which may be due to small sample sizes or insufficient study design.

This study included 17 randomized controlled trials (739 older adults), all of which assessed cognitive function. The results indicated that resistance training significantly improves cognitive function, particularly enhancing working memory, spatial memory breadth, and verbal learning and memory in older adults. These findings align with previous meta-analytic results. For instance, Helio et al. (36) conducted a meta-analysis of 18 randomized controlled trials examining the effects of resistance training on total cognitive function in older adults with varying cognitive statuses, revealing significant improvements in both cognitively healthy and cognitively impaired older adults. Similarly, Northey (3) found that resistance training plays a crucial role in enhancing cognitive function among older adults in their meta-analysis of 39 different exercise modalities, with particularly notable effects on working memory. Mechanistically, resistance training may promote neuroplasticity and increase brain volume (37). This is achieved through the enhancement of brain-derived neurotrophic factor (BDNF) release, a protein closely associated with neuronal survival, development, and synaptic plasticity. In older adults, increased levels of brain-derived neurotrophic factor (BDNF) are considered strong evidence of better cognitive function and memory performance. Resistance training enhances neuroplasticity in the hippocampus, thereby improving memory-related cognitive functions. Furthermore, resistance training can enhance functional connectivity among various brain regions, particularly within networks involved in attention and executive function. These changes in connectivity contribute to improved integrative capabilities of the brain, leading to enhanced cognitive function (38). In this study, We found that resistance training demonstrated significant positive effects on working memory in older adults, including improvements in spatial memory span and verbal learning and memory. This may be attributed to resistance training's ability to increase gray matter density in the hippocampus, thereby enhancing spatial memory capabilities (39–41), Additionally, resistance training may improve verbal learning and memory by enhancing cardiovascular function and metabolic levels in older adults (14, 42), Furthermore, resistance training promotes neuroplasticity and the production of neurotrophic factors, which can strengthen memory function (43–45). The combined effects of these factors may lead to improvements in spatial memory breadth and verbal learning and memory capabilities in older adults. Moreover, we found that resistance training did not have a significant impact on executive function, attention, or information processing speed in older adults. This finding contrasts with the results of a meta-analysis by Kelly (46), which reported positive effects of resistance training on cognitive function in older adults, including improvements in information processing speed. Supporting this discrepancy, Liu (11) also conducted a randomized controlled trial that found no significant effect of resistance training on information processing speed in older adults. We attribute these differences to factors such as the measurement tools used for outcome indicators and the quality of the included literature. Another possibility is that our analysis primarily included healthy, community-dwelling older adults, whereas previous meta-analyses incorporated individuals who were frail, had mild cognitive impairment, or exhibited clinical cognitive deficits. Such baseline differences in cognitive and physical status may influence responsiveness to resistance training, which could partly explain the variations in the magnitude of cognitive benefits observed across studies. Based on this, Our evidence indicates that resistance training is a crucial intervention for slowing cognitive decline in older adults. Compared to other forms of exercise, resistance training places less physical stress on older individuals and is generally easier to master and practice, requiring neither high-intensity efforts nor extensive cardiovascular endurance. This makes resistance training one of the more sustainable forms of exercise for older adults, facilitating cognitive improvements.

Subgroup analysis based on age revealed that the cognitive benefits of resistance training were more pronounced in individuals aged 65–74, with limited effects observed in those over 75.Cognitive reserve, which serves as a “buffering mechanism” against aging and neurodegeneration, is generally established by age 65–74, providing resilience against cognitive decline associated with aging and disease (47), Resistance training may enhance cognitive reserve by improving cerebral blood flow and neuroplasticity (48), allowing individuals within this age range to perform better on complex tasks. However, in those aged 75 and older, cognitive reserve diminishes progressively, reducing the buffering capacity over time (47). Consequently, while resistance training can help mitigate cognitive decline, its effects on cognitive improvement are less pronounced in older adults over 75 due to a lower reserve capacity (48).

Exercise dosage is a crucial variable modulating the cognitive benefits of resistance training in older adults. Subgroup analyses of the five exercise components suggest that resistance training of at least 12 weeks, conducted three times weekly for 30–60 minutes per session, significantly enhances overall cognitive function in the elderly. Due to inconsistent evidence across studies, this analysis did not stratify by exercise intensity; however, research indicates that both high- and low-intensity exercise improve cognitive function without significant differences between them (49). According to foundational principles of exercise training (15, 50) resistance training should follow a progressive overload model, where intensity or load is gradually increased to promote adaptations in both muscle and neural systems. In older adults, prolonged resistance training facilitates progressive adaptation to higher loads, with cognitive benefits gradually emerging. Training periods exceeding 12 weeks provide sufficient time for neuroplasticity and brain function adaptations, supporting structural remodeling in cognition-related areas, such as the hippocampus. Additionally, a regimen of resistance training three times weekly aligns with the principles of frequency and consistency, essential for sustaining adaptive responses in the elderly. Maintaining this frequency fosters cumulative effects on the nervous and muscular systems, enhancing cerebral blood flow and neuroplasticity. Sessions lasting 30–60 minutes are sufficient to stimulate cognitive areas without causing excessive fatigue, allowing for ample neuronal activation and recovery time. Adequate rest, particularly crucial for older adults, promotes the formation and stabilization of neural connections, thereby aiding cognitive function improvement. In sum, based on the principles of specificity and individualization, resistance training at a frequency of three times weekly, with sessions lasting 30–60 minutes over a period of 12 weeks or longer, offers an effective balance between neural adaptation and fatigue management, optimizing brain regions tied to cognitive function. Adjustments in exercise intensity should further consider individual health status and fitness goals.

This study has several limitations:1.Some included studies involved combined exercise and medication interventions, making it difficult to isolate the effects of exercise alone, which may impact the study results. 2.Despite sensitivity and subgroup analyses, the source of heterogeneity in overall cognitive function was not identified. The low heterogeneity observed may be due to the limited number of studies rather than true homogeneity, potentially affecting the reliability and generalizability of the conclusions. 3.Potential publication bias was identified, suggesting that the observed effects might be overestimated and should be interpreted with caution. 4.The limited number of randomized controlled trials assessing spatial memory span and verbal learning and memory in older adults warrants caution in interpreting these findings. 5.Only publicly available English-language studies were included, potentially introducing language bias. 6.Cognitive function measurement methods varied across studies, and the number of studies using identical outcome indicators was limited, which may affect the comparability and synthesis of results. 7.Research on the specific effects of the five elements of exercise, as well as the optimal intensity and modalities for improving cognitive function in older adults, remains scarce, highlighting the need for further investigation.

## 5 Conclusion

Resistance exercise demonstrates selective positive effects on cognitive function in older adults, with significant improvements observed in overall cognitive function, working memory, spatial memory span, and verbal learning and memory. The extent of these improvements is influenced by age and displays a dose-response relationship with intervention type, duration, frequency, and cycle. Evidence suggests that resistance training conducted three times per week, for 30–60 minutes per session, over a period of 12 weeks or more, may be an effective exercise strategy for enhancing overall cognitive function in older adults.

## Data Availability

The original contributions presented in the study are included in the article/**Supplementary Material**. Further inquiries can be directed to the corresponding author.
