# Supplementary material for: A systematic review and meta-analysis of the effects of resistance exercise on cognitive function in older adults
Source: Front Psychiatry. 2025 Dec 19;16:1708244. doi: 10.3389/fpsyt.2025.1708244 (PMC12772445; doi:10.3389/fpsyt.2025.1708244)
Supplement: Supplementary file 1 [file Table1.pdf]

Database	Search Date	Search Fields / Syntax	Representative Search String
**PubMed**	September 2024	Title/Abstract + MeSH	("resistance exercise"[Title/Abstract] OR "strength training"[Title/Abstract] OR "weight exercise"[Title/Abstract]) AND ("cognitive function"[Title/Abstract] OR "cognitive performance"[Title/Abstract] OR "executive function"[Title/Abstract] OR "working memory"[Title/Abstract] OR "inhibition"[Title/Abstract] OR "task switching"[Title/Abstract]) AND ("oldest"[Title/Abstract] OR "older adults"[Title/Abstract] OR "elderly"[Title/Abstract] OR "geriatric"[Title/Abstract] OR "aging"[Title/Abstract] OR "older people"[Title/Abstract])
**Web of Science**	September 2024	Topic (TS)	TS=("resistance exercise" OR "strength training" OR "weight exercise") AND TS=("cognitive function" OR "cognitive performance" OR "executive function" OR "working memory" OR "inhibition" OR "task switching") AND TS=("oldest" OR "older adults" OR "elderly" OR "geriatric" OR "aging" OR "older people")
**Science Direct**	September 2024	Title/Abstract/Keywords	("resistance exercise" OR "strength training" OR "weight exercise") AND ("cognitive function" OR "cognitive performance" OR "executive function" OR "working memory" OR "inhibition" OR "task switching") AND ("oldest" OR "older adults" OR "elderly" OR "geriatric" OR "aging" OR "older people")
**Embase**	September 2024	Title/Abstract	('resistance exercise'/exp OR 'strength training'/exp OR 'weight exercise'/exp OR 'resistance exercise':ti,ab OR 'strength training':ti,ab OR 'weight exercise':ti,ab) AND ('cognitive function'/exp OR 'cognitive performance'/exp OR 'executive function'/exp OR 'working memory'/exp OR 'inhibition'/exp OR 'task switching'/exp OR 'cognitive function':ti,ab OR 'cognitive performance':ti,ab OR 'executive function':ti,ab OR 'working memory':ti,ab OR 'inhibition':ti,ab OR 'task switching':ti,ab) AND ('older adult'/exp OR 'elderly'/exp OR 'aging'/exp OR 'older people'/exp OR 'oldest':ti,ab OR 'older adults':ti,ab OR 'elderly':ti,ab OR 'geriatric':ti,ab OR 'aging':ti,ab OR 'older people':ti,ab)
